# Pharmacy in America

**Published:** 1883-10-20

**Authors:** 


### PHARMACY IN AMERICA.

In respect to the beauty, variety and excellency of Pharmaceutical preparations, and the enterprise displayed by the large manufacturing chemists of this country, it is conceded that America is in advance of our transatlantic brethren.

At the late International Pharmaceutical Exhibition at Vienna, it is said that this fact was manifestly apparent.

A correspondent of the Monthly Magazine of Pharmacy thus writes of the display made by our enterprising and renowned countrymen, Messrs. Parke, Davis & Co., of Detroit:

"Since I have mentioned America, I should not forget to say that Messrs. Parke, Davis & Co., of Detroit, have an important exhibit here, in which we find represented almost all that the apothecary's art can do in America. In the absence of important exhibits by English and French houses, the Germans are evidently pleased at the extent and beauty of the American display. A great portion of it is devoted to newer American drugs, which are as yet very little known in Europe, and are classed as raw products from which the pure active principles have not yet been extracted."

"We are pleased to note that the same firm have been awarded the gold medal of the First Viennese International Pharmaceutical Exhibition, held at the Austrian capital from August 11th to 28th, inclusive."

